# Prognostic value of X-chromosome inactivation in symptomatic female carriers of dystrophinopathy

**DOI:** 10.1186/1750-1172-7-82

**Published:** 2012-10-23

**Authors:** Jonàs Juan-Mateu, Maria José Rodríguez, Andrés Nascimento, Cecilia Jiménez-Mallebrera, Lidia González-Quereda, Eloy Rivas, Carmen Paradas, Marcos Madruga, Pedro Sánchez-Ayaso, Cristina Jou, Laura González-Mera, Francina Munell, Manuel Roig-Quilis, Maria Rabasa, Aurelio Hernández-Lain, Jorge Díaz-Manera, Eduard Gallardo, Jordi Pascual, Edgard Verdura, Jaume Colomer, Montserrat Baiget, Montse Olivé, Pia Gallano

**Affiliations:** 1 Servei de Genètica, Hospital de la Santa Creu i Sant Pau and CIBERER, Barcelona, Spain; 2 Universitat de Barcelona (UB), Barcelona, Spain; 3 Unitat de Patologia Neuromuscular, Servei de Neurologia, Hospital Sant Joan de Déu, Barcelona, Spain; 4 Servicio de Anatomía Patológica, Hospital Universitario Virgen del Rocío, Sevilla, Spain; 5 Unidad de Enfermedades Neuromusculares, Servicio de Neurología, Hospital Universitario Virgen del Rocío, Sevilla, Spain; 6 Unidad de Neurología Pediátrica, Hospital Universitario Virgen del Rocío, Sevilla, Spain; 7 Servicio de Neurología, Hospital General Universitario de Albacete, Albacete, España; 8 Servei d’Anatomia Patològica, Hospital Sant Joan de Déu, Barcelona, Spain; 9 Servei de Neurologia, Hospital de Viladecans, Barcelona, Spain; 10 Institut de Recerca Hospital Universitari Vall d'Hebrón, Barcelona, Spain; 11 Secció de Neurología Infantil, Hospital Materno-Infantil, Hospital Universitari Vall d'Hebron, Barcelona, Spain; 12 Neurología, Hospital Universitario de Fuenlabrada, Fuenlabrada, Spain; 13 Servicio de Neuropatología, Hospital Universitario 12 de Octubre, Madrid, Spain; 14 Laboratori de Malalties Neuromusculars, Servei de Neurologia, Hospital de la Santa Creu i Sant Pau, Institut de Recerca de HSCSP, Universitat Autònoma de Barcelona and CIBERNED, Barcelona, Spain; 15 Servei de Neurologia, Hospital del Mar and Universitat Autònoma, Barcelona, Spain; 16 Institut de Neuropatologia, IDIBELL-Hospital de Bellvitge and CIBERNED, Hospitalet de Llobregat, Catalonia, Spain

**Keywords:** Dystrophin, *DMD*, Symptomatic carrier, Duchenne muscular dystrophy, Becker muscular dystrophy, X-chromosome inactivation

## Abstract

**Background:**

Between 8% and 22% of female carriers of *DMD* mutations exhibit clinical symptoms of variable severity. Development of symptoms in *DMD* mutation carriers without chromosomal rearrangements has been attributed to skewed X-chromosome inactivation (XCI) favouring predominant expression of the *DMD* mutant allele. However the prognostic use of XCI analysis is controversial. We aimed to evaluate the correlation between X-chromosome inactivation and development of clinical symptoms in a series of symptomatic female carriers of dystrophinopathy.

**Methods:**

We reviewed the clinical, pathological and genetic features of twenty-four symptomatic carriers covering a wide spectrum of clinical phenotypes. *DMD* gene analysis was performed using MLPA and whole gene sequencing in blood DNA and muscle cDNA. Blood and muscle DNA was used for X-chromosome inactivation (XCI) analysis thought the *AR* methylation assay in symptomatic carriers and their female relatives, asymptomatic carriers as well as non-carrier females.

**Results:**

Symptomatic carriers exhibited 49.2% more skewed XCI profiles than asymptomatic carriers. The extent of XCI skewing in blood tended to increase in line with the severity of muscle symptoms. Skewed XCI patterns were found in at least one first-degree female relative in 78.6% of symptomatic carrier families. No mutations altering XCI in the *XIST* gene promoter were found.

**Conclusions:**

Skewed XCI is in many cases familial inherited. The extent of XCI skewing is related to phenotype severity. However, the assessment of XCI by means of the *AR* methylation assay has a poor prognostic value, probably because the methylation status of the *AR* gene in muscle may not reflect in all cases the methylation status of the *DMD* gene.

## Background

The dystrophinopathies are a group of X-linked muscle diseases caused by mutations in the *DMD* gene. Clinical phenotypes vary from asymptomatic high CK levels and cramps to severe progressive skeletal and cardiac muscle disorders such as Duchenne (DMD) and Becker (BMD) muscular dystrophies, and X-linked dilated cardiomyopathy (XLCM) [1]. About one third of patients present mental retardation [2]. Most disease-responsible mutations are large intragenic rearrangements (exonic deletions and duplications) that account for 65 to 75% of cases, while the remaining cases are caused by single point mutations or small rearrangements [3,4]. In most patients, the clinical outcome can be predicted according to the reading-frame rule. The majority of DMD patients carry truncating mutations while BMD patients usually carry in-frame mutations allowing the expression of semi-functional dystrophins [1,5].

Female carriers of *DMD* mutations are usually asymptomatic due to the X-linked inheritance of the disease. However, symptomatic carriers can manifest a wide spectrum of clinical symptoms ranging from myalgia and cramps on exertion to severe disabling DMD-like muscle weakness [6-11]. Onset of symptoms fluctuates from early childhood to the fourth decade. The percentage of carriers with clinical abnormalities varies among the series [7,12]. Hoogerwaard et al. found that 5% present regular myalgia and cramps without muscle weakness, 17% show mild to moderately severe muscle weakness, and 8% present dilated cardiomyopathy, showing an average onset at 33 years [12].

Several disease-causing mechanisms have been implicated in DMD/BMD manifesting carriers. These include X-autosomal translocations disrupting the *DMD* gene [13], mutations on both *DMD* alleles [10,14] and co-occurrence of *DMD* mutations together with other genetic abnormalities such as X-chromosome monosomy [15-17], X-chromosome uniparental disomy [18] as well as male pseudohermaphroditism caused by a mutation in the androgen receptor gene [19]. However, the most frequently reported mechanism to provoke symptoms in DMD carriers is skewed X-inactivation, favouring the expression of the X chromosome with the *DMD* mutated allele [8,20-22]. Although some studies suggest the use of X-inactivation analysis for prognostic purposes, the results of different reports are controversial [21,23,24].

In this study we aimed to investigate the prognostic value of X-chromosome inactivation in a large series of dystrophinopathy affected females presenting with a wide spectrum of clinical phenotypes.

## Materials and methods

### Patients

We reviewed our database records of all dystrophinopathy patients. The database includes patients with a clinical history compatible with dystrophinopathy, X-linked family history of myopathy and/or a muscle biopsy showing abnormal dystrophin immunostaining. We identified 24 symptomatic carrier females referred from different centres around Spain. For the present study we included females with a confirmed *DMD* mutation or a muscle biopsy showing altered dystrophin staining, who manifest at least one of the following symptoms: myalgia, dilated cardiomyopathy, cognitive abnormalities or muscle weakness. We retrospectively collected data regarding clinical features, serum CK levels, cardiological studies and muscle biopsy analysis. Patients were grouped into different phenotype categories on the basis of their clinical course.

Two control groups were selected for X-chromosome inactivation (XCI) studies. The first control group consisted of 40 asymptomatic female carriers who presented 28 different *DMD* mutations: 20 exonic deletions, 5 exonic duplications, and 3 point mutations. The second control group included 41 confirmed non-carrier females from 28 unrelated DMD/BMD families with known *DMD* mutations. The study was approved by the Ethical Committe of Hospital de la Santa Creu i Sant Pau (HSCSP), Barcelona. All participants gave their written informed consent.

### Muscle biopsy pathological analysis

A muscle biopsy was taken in 17 of the 24 symptomatic carriers. These were processed for routine histological and histochemical techniques, and for dystrophin immunohistochemistry as described elsewhere [25].

### Genetic analysis

DNA extracted from peripheral blood underwent *DMD* mutational analysis using a combination of techniques. Intragenic deletions and duplications were analyzed using MLPA (P034 and P035 Salsa Kit, MRC-Holland). Point mutation detection was done by whole gene sequencing using published primers [26]. When a muscle biopsy was available, total mRNA was extracted and purified from approximately 30 mg of muscle using RNeasy Fibrous Tissue Mini Kit (Qiagen, Hilden, Germany). Subsequently, muscle mRNA was retrotranscribed to cDNA by RT-PCR using polythymine primers (Invitrogen, Carlsbad, NM). Complete *DMD* cDNA was amplified and sequenced in twenty overlapping fragments using published [27] and self-designed primers. Splicing mutations were analyzed using predictive software (Human Splice Finder and NNSPLICE). Nucleotide positions were determined according to the standard *DMD* reference sequence (GenBank accession number NM_004006.2). In eight families segregation analysis of the Xp21 locus was performed using microsatellite markers: 5’DYS-1, DXS1242, DXS1243, DXS206, DXS1238, DXS1237, DXS1236, DXS1235 and DXS1234. In four cases (subjects #1, #2, #18 and #19) a karyotype was performed before the *DMD* molecular analyses; all four cases showed a normal 46XX karyotype.

### X-chromosome inactivation (XCI) analysis

Methylation of the *Hpa*II restriction site near a polymorphic (CAG)_n_ repeat in the *AR* gene (Androgen Receptor) correlates with XCI [28]. We used *Hpa*II digestion followed by QF-PCR to determine the methylation status of parental X chromosomes. Active alleles are digested while the inactive alleles are not. The ratio of undigested parental alleles gives the pattern of X-chromosome inactivation.

For each sample, 500 ng of DNA were digested with *Hpa*II restriction endonuclease (New England Biolabs, Ipswich, MA). Digested products together with non-digested DNA were used as templates for amplification of the *AR* polymorphic repeat using fluorescence labelled primers. PCR fragments were run in an ABI 3500xL Genetic Analyzer (Applied Biosymtems, Foster City, CA). *AR* alleles were sized and quantified using Genemapper software (Applied Biosymtems, Foster City, CA). To correct preferential allele amplification, the allele ratio in *Hpa*II digested DNA was normalized using the ratio of non-digested DNA. Monoallelic patients for the *AR* repeat were reported as “non-informative”. Following previously published criteria, XCI ratios equal to or less than 80:20 were considered “random” patterns while ratios greater than 80:20 were considered “skewed” patterns [29].

XCI studies were performed in lymphocyte DNA from 24 symptomatic carriers, 40 asymptomatic carriers and 41 non-carrier females. To detect familial XCI skewing, analyses were also performed in at least one first-degree female relative (mother, daughter or sister) in 15 of the 23 families with a symptomatic carrier. In 9 symptomatic carriers XCI analysis was also performed on muscle DNA.

### *XIST* minimal promoter mutation analysis

The expression of the non-coding gene *XIST* is involved in the mechanisms that determine the choice of inactive X chromosome [30]. Mutations c.-43C > A and c.-43C > G, located in the *XIST* promoter region have been associated to cause XCI skewing [31,32]. *XIST* minimal promoter was amplified and sequenced in order to detect XCI altering mutations using forward primer 5’ACCCATTGAAGTTGTGACTCCTGGT3’ and reverse primer 5’ACGCCATAAAGGGTGTTGGGGG3’.

### Statistical analysis

Statistical comparison of proportion of skewed XCI between two groups was estimated using Fisher's Exact Test. Comparison of XCI ratios between two groups was estimated using paired t test while comparison among groups was done using one-way ANOVA, followed by the Bonferroni post hoc test. P values lower than 0.05 were considered as statistically significant.

## Results

### Clinical presentation

From a total of 344 female carriers of *DMD* mutations we identified 24 (7%) patients from 23 unrelated families who presented symptoms associated with dystrophinopathy. Two cases were identified within the same family (subject #13, mother, and subject #14, daughter). Clinical features are summarized in Table 1. Clinical presentations were highly heterogeneous and included: isolated dilated cardiomyopathy (*n* = 2, 8.3%), isolated cognitive abnormalities (*n* = 3, 12.5%), myalgia without muscle weakness (*n* = 4, 16.7%), and mild to severe muscle weakness (*n* = 15, 62.5%). Patients presenting muscle weakness were grouped into three different phenotype categories: mild BMD-like (*n* = 7, 29%), severe BMD-like (*n* = 5, 21%) and DMD-like (*n* = 2, 8.3%). Subject #3 presented with weakness at the age of 2 years and was too young to be assigned to either DMD-like or severe BMD-like. Muscle weakness was asymmetric in 6 cases (subjects #6, #9, #10, #13, #14 and #15). Age of onset varied from 2 to 74 years (median: 17.5; mean: 21.7). The most common presenting symptom was muscle weakness (*n* = 14, 58.3%) followed by myalgia, cramps and/or exercise intolerance (*n* = 8, 33.3%).

**Table 1:** **Clinical features of symptomatic carriers of*DMD*mutations**

**Patient**	**Age of onset**	**Phenotype**	**Presenting symptoms**	**Age at most recent exam**	**Clinical symptoms at most recent exam**	**CK levels (age)**	**Affected relative**
1	2	DMD-like	Global developmental delay, weakness	13	Severe mental retardation, severe weakness, wheelchair-bound at 13, normal echocardiogram	22055 (13)	no
2	4	DMD-like	Weakness, calf pseudohypertrophy	14	Severe weakness, wheelchair-bound at 10, normal echocardiogram	12000 (4)	no
3	2	D/BMD-like	Frequent falls, seizures	5	Mild weakness in lower limbs, normal echocardiogram	n/p	no
4	12	Severe BMD-like	Weakness	29	Severe weakness and atrophy	n/p	DMD
5	4	Severe BMD-like	Exercise intolerance, weakness, poor school performance	12	Incomplete Gower's sign, herculean appearance, calf pseudohypertrophy, mild mental retardation	7678-333 (8–12)	no
6	7	Severe BMD-like	Asymmetric weakness	11	Asymmetric weakness, more severe in shoulder than in pelvic girdle, normal echocardiogram	n/p	no
7	20	Severe BMD-like	Weakness	24	Severe weakness of upper and lower limbs, need a wheelchair for long walks, normal echocardiogram	1700 (24)	no
8	4	Severe BMD-like	Frequent falls, weakness	26	Severe weakness, walks with mayor difficulties, calf pseudohypertrophy, normal echocardiogram	5331 (22)	no
9	28	Mild BMD-like	Asymmetric weakness	32	Moderate unilateral weakness of right limbs, normal echocardiogram	1500 (32)	DMD
10	41	Mild BMD-like	Weakness, calf pseudohypertrophy	42	Mild weakness, asymmetric in shoulder, asymmetric atrophy of posterior leg compartment	6654 (42)	DMD
11	18	Mild BMD-like	Myalgia/cramping, calf pseuhypertrophy	49	Moderate muscle weakness, normal echocardiogram	1527 (49)	DMD
12	30	Mild BMD-like	Myalgia, weakness	38	Moderate muscle weakness, normal echocardiogram	1288 (30)	DMD
13	31	Mild BMD-like	Finding elevated CK prompted a neurological examination revealing mild weakness	51	Moderate asymmetric weakness	2857 (31)	DMD
14	13	Mild BMD-like	Detecting weakness in her mother prompted neurological examination finding mild weakness	33	Moderate asymmetric weakness, calf pseuhypertrophy	10530-6950 (13–33)	DMD
15	39	Mild BMD-like	Frequent falls, myalgia/cramping, weakness	51	Moderate asymmetric weakness, more affected in lower limbs, mild non-specific echocardiographic changes and bundle branch block	1770-590 (43–51)	DMD
16	66	DCM	Dilated cardiomyopathy with severe ventricular dysfunction	69	Dilated cardiomyopathy with no muscle weakness	n/p	DMD
17	74	DCM	Dilated cardiomyopathy	77	Dilated cardiomyopathy with no muscle weakness	n/p	DMD
18	7	Behavioural issues	Abnormal behaviour, elevated CK levels	12	Behavioural abnormalities without mental retardation, no muscle weakness, normal echocardiogram	3000 (7)	BMD
19	4	MR	Delayed speech development, abnormal behaviour, elevated CK levels	8	Mild mental retardation, no muscle weakness	1137 (4)	no
20	4	MR	Learning difficulties, elevated CK levels	18	Mild mental retardation, no muscle weakness	1193 (14)	no
21	31	Myalgia	Precordial pain, myalgia	36	Regular myalgia, no muscle weakness, normal echocardiogram	897 (36)	DMD
22	33	Myalgia	Myalgia	34	Regular myalgia, no muscle weakness	450 (34)	DMD
23	17	Myalgia	Exercise intolerance	19	Regular myalgia, no muscle weakness	n/p	no
24	30	Myalgia	Myalgia	32	Regular myalgia, no muscle weakness	n/p	no

Patient #3, D/BMD-like, was too young to be assigned to either DMD-like or severe BMD-like phenotypes. DCM: dilated cardiomyopathy, MR: mental retardation.

Cognitive abnormalities were found, in isolation or together with muscle weakness, in 5 cases (20.8%). These abnormalities ranged from behavioural issues to severe global developmental delay. Subject #1, who presented a severe DMD-like phenotype, showed severe cognitive impairment with comprehensive and expressive language almost absent. Subjects #5, #19 and #20 presented with learning difficulties or poor academic performance showing mild mental retardation in a WISC-IV test, while subject #18 presented poor social and communication skills but no mental retardation. In subjects #18, #19 and #20, cognitive abnormalities were the only clinical symptoms. In these subjects, other aetiologies of mental retardation such as metabolic diseases, brain malformations, chromosomal disorders or fragile X syndrome were considered. These were ruled out by hormone and metabolic profiling in blood and urine (subjects #18, #19 and #20), neuroimaging studies (subjects #19 and #20), karyotype analysis (subjects #18 and #19) and *FMR1* molecular analysis (subject #19). Subject #18 presented a BMD affected brother while in subjects #19 and #20, in absence of previous family history of neuromuscular disease, elevation of CK levels or a muscle biopsy showing abnormal dystrophin expression prompted *DMD* molecular studies. We could not exclude that the behavioural abnormalities of subject #18 were aetiologically independent of the dystrophinopathy. However, her BMD affected brother presented mild muscle symptoms and mental retardation with autistic behaviour indicating that the behavioural issues could be related to the dystrophinopathy.

Echocardiographic studies were performed in 14 cases and abnormalities were detected in three (21.4%). Severe cardiac dysfunction caused by dilated cardiomyopathy (DCM) was found in two cases (subjects #16 and #17) that did not present accompanying muscle symptoms. Onset of symptoms in these two patients was 66 and 74 years. Subject #15 showed mild non-specific echocardiographic abnormalities and a bundle branch block on EKG but no signs of cardiac failure.

### Muscle biopsy pathological findings

**Figure 1:**
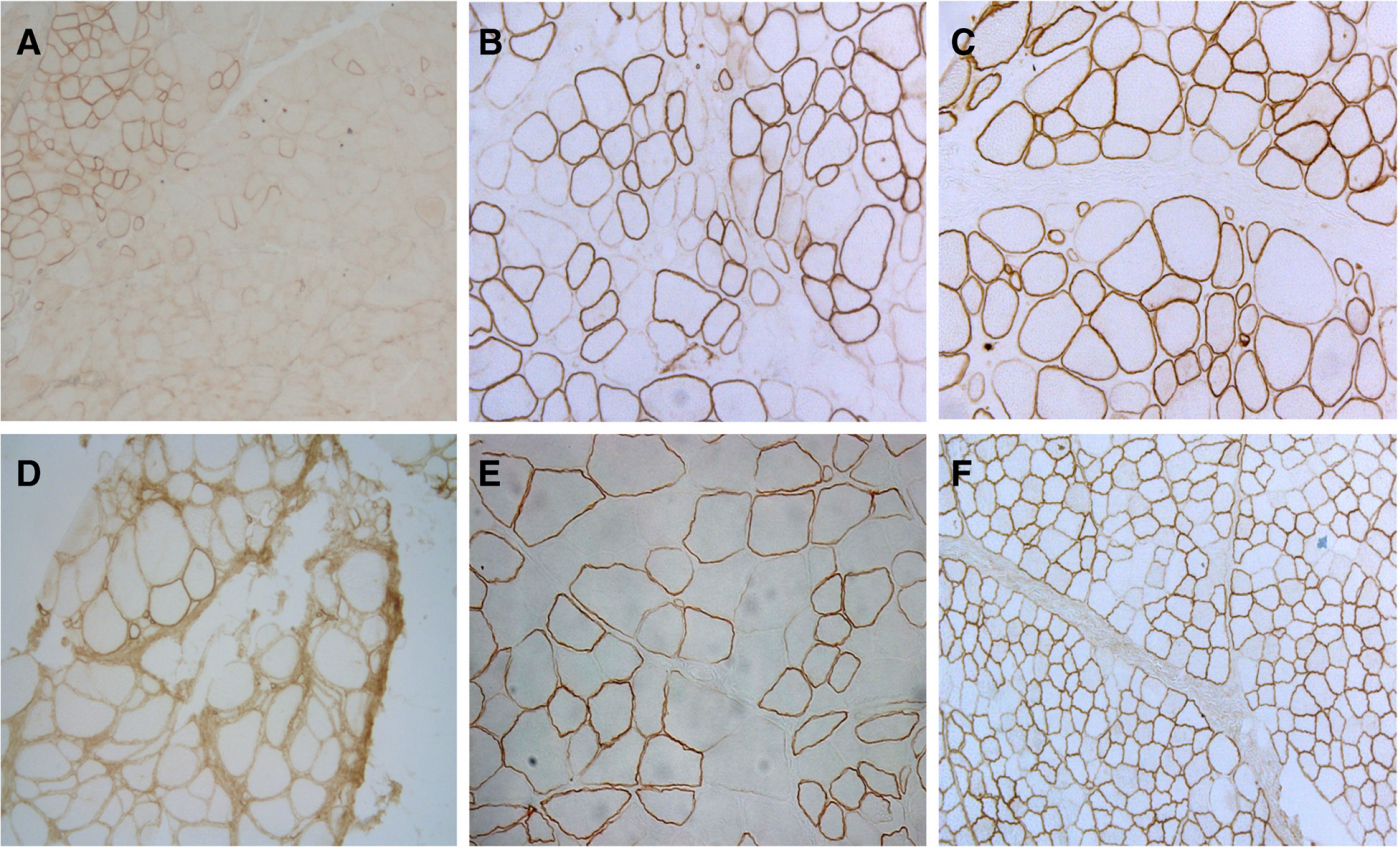
**Dystrophin immunostaining in muscle biopsy sections from representative symptomatic carriers.A**) D/BMD-like subject #3, **B**) severe BMD-like subject #4, **C**) severe BMD-like subject #6, **D**) severe BMD-like subject #8, **E**) mild BMD-like subject #13, **F**) subject #19 presenting mild mental retardation but no muscle weakness. No association was found between dystrophin abnormalities and clinical phenotype.

Muscle biopsy was performed in 17 cases. Results are summarized in Table 2 and Figure 1. Abnormal pathological features were found in all analyzed cases, and included variation in fibre size, increased numbers of internal nuclei, muscle fibre necrosis and regeneration, and variable degree of endomysial fibro-fatty tissue proliferation. Non-specific myopathic changes were detected in four patients, whereas mild to severe dystrophic changes were found in the remaining thirteen. Immunohistochemical analysis revealed a mosaic pattern of dystrophin-positive and reduced or absent dystrophin fibres in 9 cases (53%). In 4 cases only isolated dystrophin-negative fibres were observed. Normal dystrophin staining was found in a single patient (#18) who presented behavioural issues as the only symptom. Generalized absence of dystrophin expression was observed in two patients (subjects #1 and #3), both of whom suffered from muscle weakness from early childhood.

**Table 2:** **Summary of genetic findings and muscle biopsy features in symptomatic carriers of*DMD*mutations**

**Patient**	**Phenotype**	***DMD*mutation**	**Muscle biopsy features**	**Dystrophin immunostaining**	**Blood DNA XCI**	**Muscle DNA XCI**	**Most inactive** **X-chr.**	**Origin of*DMD*mutation**	**Familial skewed XCI**
1	DMD-like	Deletion exons 1–44 (c.(?_-244)_6438 + ?del, frameshift)	Severe dystrophic pattern	Generalized absence	**93:7**	n/p	maternal	paternal	yes
2	DMD-like	Subexonic deletion/insertion exon 17 (c.2095delinsTC, frameshift)	End-stage muscular dystrophy	n/p	**100:0**	51:49	paternal	n/p	no
3	D/BMD-like	Stop exon 8 (c.724C > T, p.Gln242X)	Severe dystrophic pattern	Generalized absence	**100:0**	61:39	paternal	n/p	yes
4	Severe BMD-like	Splice site exon 27 (c.3786 + 1G > A, predicted frameshift)	Moderate dystrophic pattern	Mosaic pattern	**94:6**	**81:19**	paternal	maternal	yes
5	Severe BMD-like	Deletion exon 68 (c.9808-?_9974 + ?del, frameshift)	Moderate dystrophic pattern	Mosaic pattern	69:31	n/p	maternal	n/p	yes
6	Severe BMD-like	Stop exon 41 (c.5893C > T, p.Gln1965X)	Severe dystrophic pattern	Reduction/absence in isolated fibres	74:26	52:48	n/i	n/p	yes
7	Severe BMD-like	Deletion exons 5–7 (c.265-?_649 + ?del, frameshift)	Severe dystrophic pattern	Mosaic pattern	**81:19**	**87:12**	n/p	n/p	n/p
8	Severe BMD-like	Deletion exon 44 (c.6291-?_6438 + ?del, frameshift)	Moderate dystrophic	Mosaic pattern with predominance of negative fibres	**100:0**	n/p	n/p	n/p	n/p
9	Mild BMD-like	Deletion exons 43–45 (c.6118-?_6614 + ?del, frameshift)	Mild dystrophic pattern	Mosaic pattern	n/i	n/p	n/p	n/p	n/p
10	Mild BMD-like	Deletion exons 45–50 (c.6439-?_7309 + ?del, frameshift)	Moderate dystrophic pattern.	Mosaic pattern	71:29	n/p	n/i	maternal	no
11	Mild BMD-like	Deletion exons 53–54 (c.7661-?_8027 + ?del, frameshift)	Mild dystrophic pattern	Reduction/absence in isolated fibres	**81:19**	n/p	n/i	maternal	no
12	Mild BMD-like	Duplication exons 50–55 (c.7201-?_8217 + ?dup, predicted in-frame)	n/p	n/p	n/i	n/p	n/p	maternal	n/p
13	Mild BMD-like	Splice site exon 48 (c.6913-1G > A, frameshift)	Mild dystrophic pattern	Mosaic pattern	**81:19**	53:47	maternal	maternal	yes
14	Mild BMD-like	Splice site exon 48 (c.6913-1G > A, frameshift)	n/p	n/p	72:28	n/p	paternal	maternal	yes
15	Mild BMD-like	Deletion exons 48–50 (c.6913-?_7309 + ?del, frameshift)	Mild dystrophic pattern	Mosaic pattern	52:48	40:60	n/p	n/p	n/p
16	DCM	Deletion exon 44 (c.6291-?_6438 + ?del, frameshift)	n/p	n/p	**99:1**	n/p	n/i	n/p	Yes
17	DCM	Deletion exons 46–52 (c.6615-?_7660 + ?del, frameshift)	n/p	n/p	63:37	n/p	n/p	n/p	n/p
18	Behavioural issues	Duplication exons 13–27 (c.1483-?_3786 + ?dup, predicted in-frame)	Myopathic changes	Normal	**99:1**	**97:3**	paternal	maternal	Yes
19	MR	Subexonic deletion exon 46 (c.6638delT, frameshift)	Myopathic changes	Absence in isolated fibres	**88:12**	69:31	paternal	maternal	yes
20	MR	Deletion exons 46–55 (c.6615-?_8217 + ?del, frameshift)	n/p	n/p	**100:0**	n/p	paternal	maternal	n/i
21	Myalgia	Duplication exons 38–43 (c.5326-?_6290 + ?dup, predicted frameshift)	n/p	n/p	74:26	n/p	paternal	maternal	yes
22	Myalgia	Deletion exons 10–43 (c.961-?_6290 + ?del, frameshift)	n/p	n/p	51:49	n/p	n/p	n/p	n/p
23	Myalgia	Deletion exon 7 (c.531-?_649 + ?del, frameshift)	Myopathic changes	Reduction/absence in isolated fibres	78:22	n/p	n/p	n/p	n/p
24	Myalgia	Deletion exons 3–13 (c.94-?_1602 + ?del, in-frame)	Myopathic changes	Mosaic pattern	50:50	n/p	n/p	n/p	n/p

X-chromosome inactivation (XCI): skewed patterns >80:20 are in bold. n/p: not performed, n/i = non-informative *AR* alleles.

### Related probands and *DMD* mutation spectrum

Among the 24 symptomatic carriers 13 had a previous family history of dystrophinopathy affected males (54.2%) while the 11 remaining cases were isolated symptomatic carriers (45.8%). The vast majority of cases with previous family history had DMD affected relatives (12/13, 92.3%). Only subject #18, who presented behavioural issues as the only clinical symptom, had a BMD affected brother. Most cases with no previous family history presented mutations predominantly associated to the DMD phenotype according to the Leiden Open Variation Database (LOVD, http://www.dmd.nl).

Twenty-two different *DMD* mutations were identified, all in heterozygous state. These are listed in Table 2 and included: 13 exonic deletions (59%), 3 exonic duplications (14%) and 6 point mutations (27%). Point mutations consisted of two nonsense mutations, two small deletions/insertions and two splicing mutations. Subjects #2, #4, #18 and #19 presented novel mutations according to LOVD. Most patients carried predicted frameshift or nonsense mutations (87.5%, 21/24). In-frame mutations included: duplication 13–27 (subject #18), not previously described, duplication 50–55 (subject #12) and, deletion 3–13 (subject #24). The latter two mutations were both associated mainly with DMD phenotype according to LOVD.

Splice site mutations were analyzed at muscle cDNA level or by predictive software in order to identify pre-mRNA splicing defects. The c.6913-1G > A mutation, identified in patients #13 and #14, a mother and daughter, destroyed the putative acceptor splice site of exon 48. At the muscle cDNA level the mutation was observed to provoke a single base deletion due to the creation of a new acceptor site at position c.6913. The c.3786 + 1G > A mutation identified in subject #4 destroyed the putative donor splice site of exon 27. Although this mutation was not analyzed at the muscle cDNA level, the presence of a cryptic donor splice site located at position c.3786 + 53 possibly provoked the retention of an intronic fragment of 52 bp.

**Figure 2:**
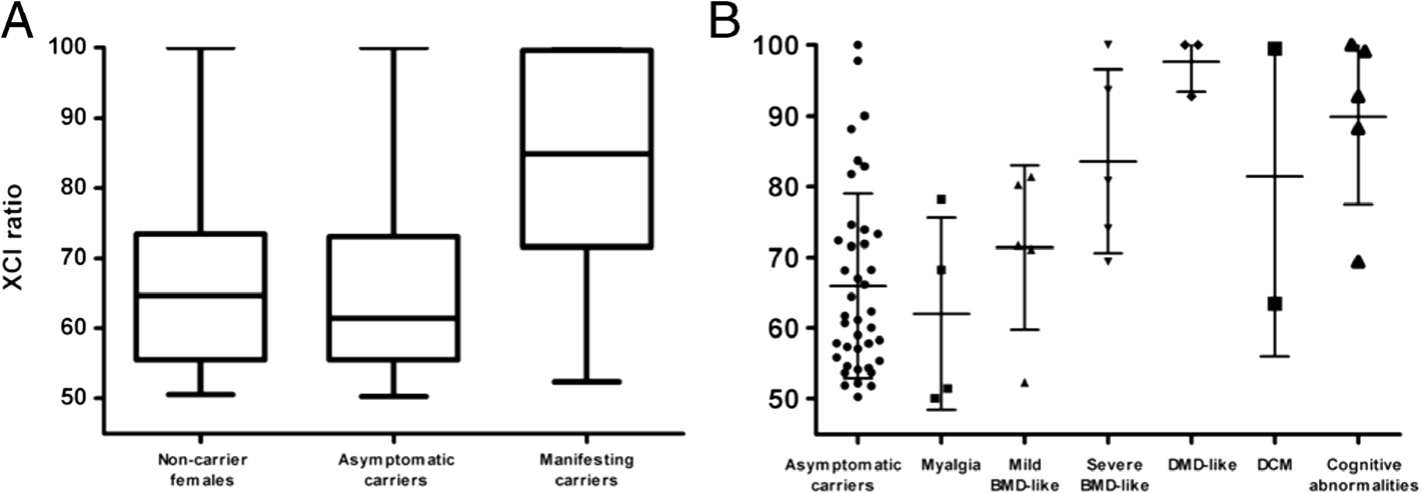
**X-chromosome inactivation patterns in blood. A**) Distribution of XCI patterns among non-carrier females (n = 41), asymptomatic carriers (n = 40) and symptomatic carriers excluding patients presenting only myalgia (n = 18). For each group, the five parameters are the lowest observation, lower quartile, median, upper quartile, and highest observation. **B**) Distribution of XCI patterns among symptomatic carriers according to clinical phenotype. Individual XCI data, mean and standard deviation are shown. The extent of XCI skewing in blood tended to increase with increasing severity of muscle symptoms.

In four cases (#3, #6, #13 and #19) mutation analysis was performed both at genomic and muscle cDNA level. In subjects #6, #13 and #19, both wild-type and mutated transcripts were detected in muscle cDNA. In subject #3, only the mutated transcript carrying a nonsense mutation was detected, while at genomic level, the mutation was in heterozygous state (Figure 2).

### X-chromosome inactivation studies (XCI)

**Figure 3:**
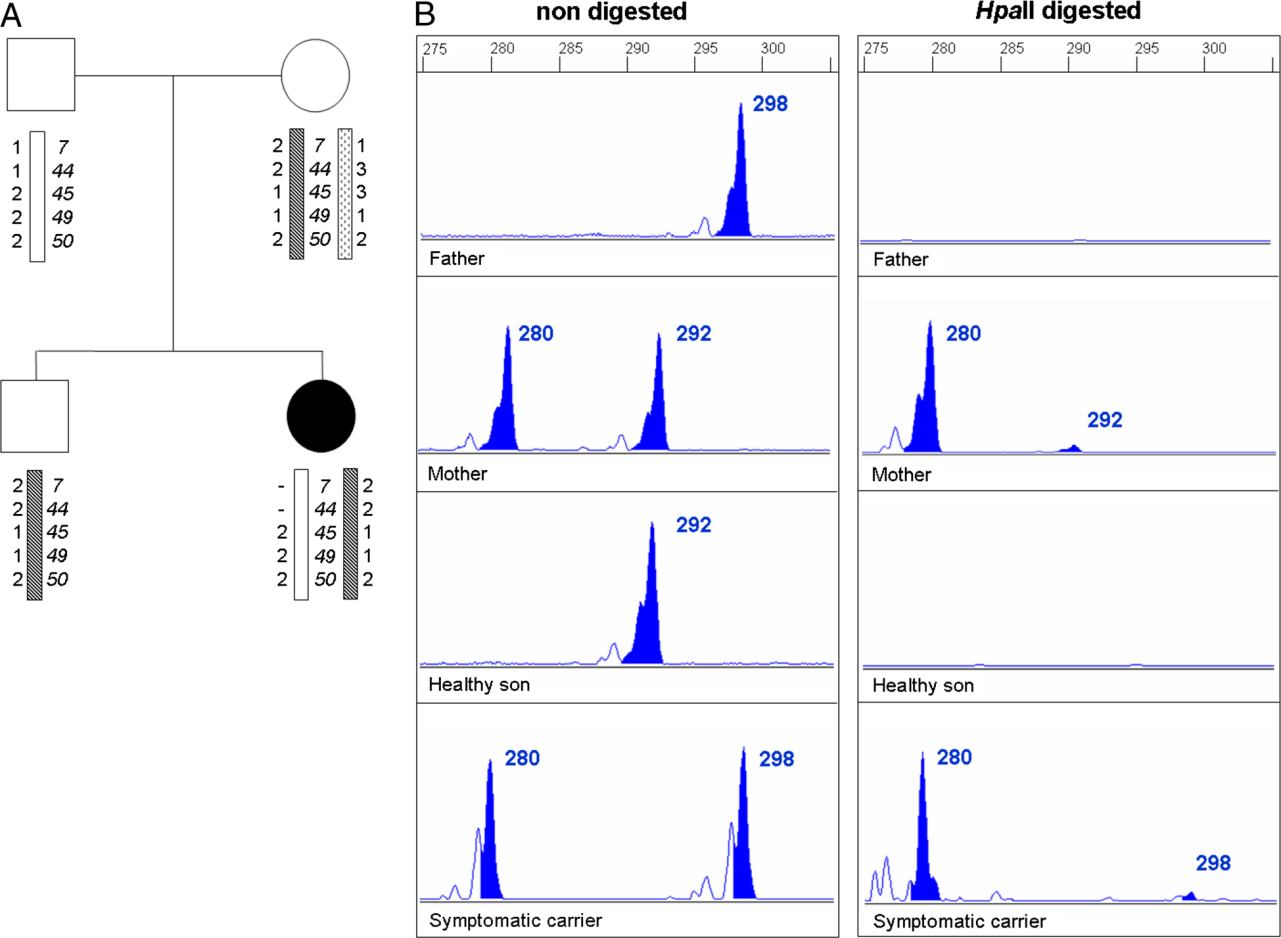
**Inheritance of *DMD *mutation, parental origin of most inactivated X-chromosome and familial skewed XCI in family of subject #1.A**) Family pedigree. Haplotype analysis of Xp21 locus indicates that the mutation is located at the paternal X-chromosome. **B**) Genemapper traces of non-digested and *Hpa*II digested *AR* alleles in blood. Methylated status of the *AR* revealed that both mother and daughter present highly skewed XCI. Maternal X-chromosome is preferentially inactivated in affected daughter indicating that the paternal X-chromosome carrying the *DMD* mutated allele is active.

XCI analysis was performed in lymphocyte DNA of the 24 symptomatic carriers, 40 non-symptomatic carriers and 41 non-carrier females. Results are summarized in Table 2 and Figure 3. XCI studies were informative in 22 symptomatic carriers. Among these, the mean XCI ratio was 80:20 ± 16.8 and a skewed XCI pattern was found in 12 cases (54.6%). If we do not consider patients presenting myalgia as a true symptomatic carriers as previously published criteria, [10,12], skewed XCI among symptomatic carriers (*n* = 18) increased to 66.7% with a mean XCI ratio of 84:16 ± 14.9. Asymptomatic carriers and non-carrier females presented similar distribution of XCI ratios: among asymptomatic carriers, 17.5% presented skewed XCI with a mean ratio of 66:34 ± 13.1, while among non-carrier females skewed XCI was found in 12.2% with a mean ratio of 66:34 ± 11.9. Differences in the XCI ratio and proportion of skewed cases between symptomatic carriers and asymptomatic carriers or non-carrier females were statistically significant (p values <0.001 by paired t-test and Fisher's Exact Test).

The extent of XCI skewing in blood tended to increase in line with the severity of symptoms among phenotype groups presenting skeletal muscle involvement (Figure 3B). While all patients with myalgia showed random XCI (mean ratio: 62:38), skewed XCI was present in 50% of cases presenting a mild BMD-like phenotype (mean ratio: 71:29), in 60% of patients with severe BMD-like phenotype (mean ratio: 84:16) and in all DMD-like cases (including subject #3, mean ratio: 98:2). However, these differences only reached statistical significance between DMD-like and myalgia groups. Skewed XCI was present in 80% of cases showing cognitive abnormalities (mean ratio 90:10). One of the two patients who presented a DCM showed a random pattern and the other showed a highly skewed pattern (mean ratio 81:19).

XCI analysis was also performed in muscle DNA in nine symptomatic carriers (Table 2). Similar XCI ratios between blood and muscle were found only in two cases, while the remaining seven exhibited significant differences. From the seven cases showing skewed XCI in blood only three presented a skewed pattern in muscle (43%). In all cases the most active X-chromosome was the same in blood and muscle.

### Parental origin of most inactivated X-chromosome and inheritance of *DMD* mutation

**Figure 4:**
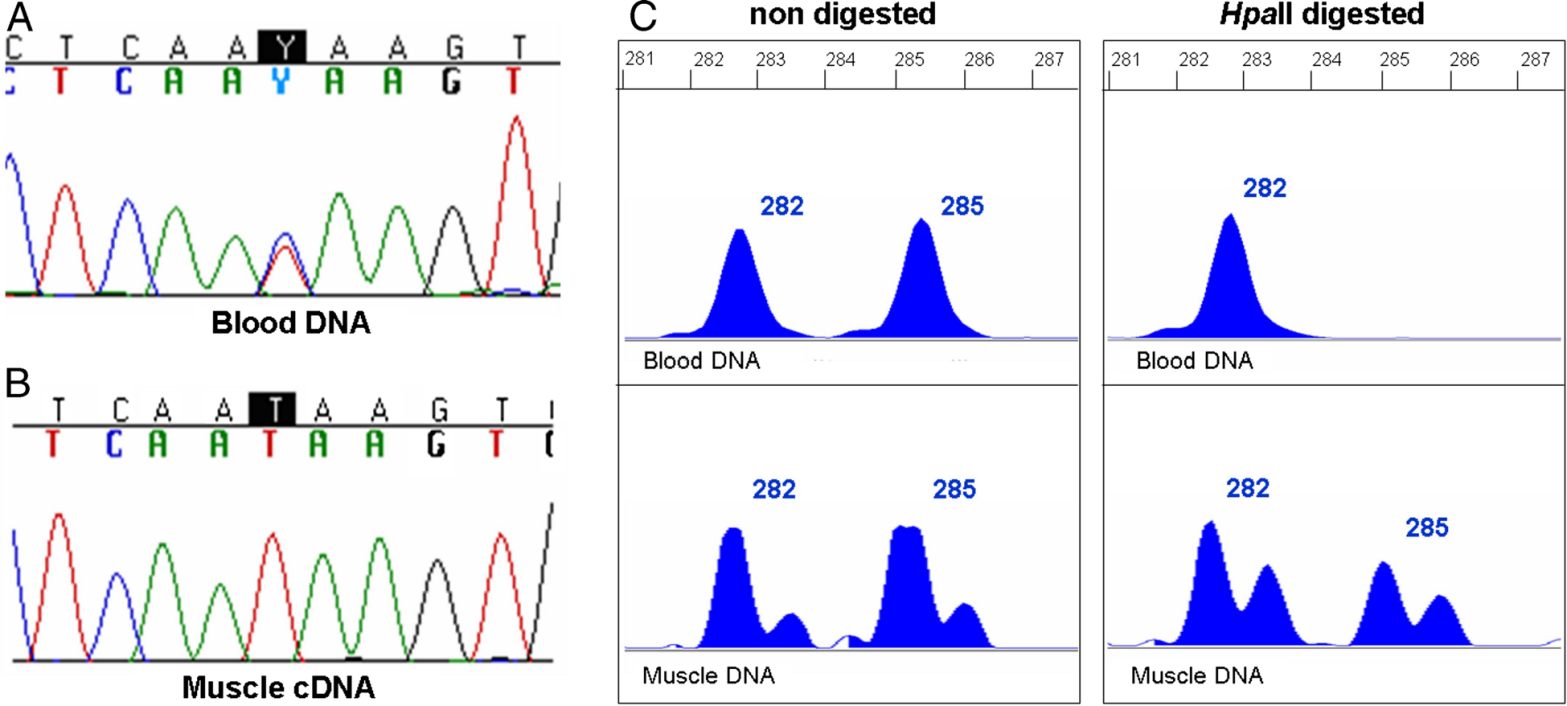
**Preferential expression of the *DMD *mutated allele correlates with blood XCI but not with muscle XCI in subject #3.A**) Automated sequence analysis of *DMD* gene exon 8 showing the nonsense mutation c.724C > T/p.Gln242X in heterozygous state. **B**) At muscle cDNA level, only the mutated transcript was detected. **C**) Genemapper traces of non-digested and *Hpa*II digested *AR* alleles in blood and muscle. Blood DNA presented a skewed XCI pattern of 100:0 while muscle DNA exhibited a random pattern of 61:39.

The parental origin of X chromosomes was determined by comparing *AR* alleles in the patient with those of her parents, while inheritance of *DMD* mutation was established either by carrier status of patient’s mother and/or by segregation analysis of Xp21 locus using polymorphic microsatellites (Table 2 and Figure 4). In most cases, most inactivated X chromosome was from paternal origin (8/11, 73%) while *DMD* mutation was inherited from the mother (10/11, 91%). In 7 of 8 cases, mutant *DMD* allele segregated with the most unmethylated *AR* allele (active X-chromosome). However, two of them did not reach a skewed XCI pattern (>80:20) in blood.

### Familial skewed X-chromosome inactivation and analysis of the *XIST* promoter

XCI studies in at least one first degree female relative (mother, daughter or sister) were performed in 15 of the 23 different families with symptomatic carriers (Figure 4). Evidence of familial skewed XCI was found in eleven of fourteen families with informative *AR* alleles (78.6%). Surprisingly, mothers of three symptomatic carriers showing random XCI in blood (subjects #5, #6 and #21) exhibited skewed XCI. The promoter region of *XIST* non-coding gene was screened in all symptomatic carriers but no mutations were detected.

## Discussion

We report the largest series of *DMD* symptomatic carriers, consisting of 24 cases covering a wide spectrum of clinical phenotypes. The main mechanism found to provoke symptoms in these patients was skewed XCI favouring expression of the *DMD*-mutated allele. We provide data consistent with the hypothesis that the extent of XCI skewing is related to phenotype severity and that skewed XCI patterns are in most cases familial inherited. We also describe what is, to our knowledge, the first report of *DMD* female carriers presenting cognitive impairment but no muscle weakness, broadening the clinical phenotypes associated with dystrophinopathy.

In line with previous reports [33], isolated symptomatic carriers were relatively frequent, suggesting that dystrophinopathy should always be considered in females suffering from a myopathy of unknown cause, even in the absence of an X-linked family history. In these cases dystrophin immunostaining on muscle sections remains the best diagnostic tool even though an exon copy number screening technique (such as MLPA) can be considered prior to muscle biopsy. Muscle biopsies showed variable pathological features in all studied cases. Furthermore, dystrophin abnormalities were found in all but one case. In accordance with previous publications [6,20,34], we did not find a correlation between dystrophin expression and clinical phenotype, although the most affected subjects showed generalized dystrophin absence (Figure 1).

Our findings confirm that symptomatic carriers present a wide clinical variability ranging from nearly asymptomatic forms to disabling DMD-like phenotypes. The progression of muscle weakness among symptomatic carriers correlates with onset of symptoms. Onset during the first decade was associated in all cases with rapid progression leading to severe phenotypes (DMD-like or severe BMD-like), while later onset, from the third decade onwards, was associated with mild progression. However, patients presenting onset of symptoms during the second decade exhibited variable severity. Subject #7 presented onset of weakness at 20 years with rapid progression leading to wheelchair support five years later, whereas subject #11 who manifested the first symptoms at 18 years is still able to walk at the age of 49 years. In accordance with previous observations [10,12,33], muscle weakness was asymmetric in 40% of our patients. Asymmetry may be caused by somatic mosaicism [35] or reflect different XCI patterns between different muscle groups and tissues. In keeping with the later hypothesis, most of our cases presenting muscle weakness and random XCI in blood showed asymmetric distribution of weakness (4/5). Five patients presented symptoms without skeletal muscle involvement: two subjects presented isolated dilated cardiomyopathy while three showed cognitive abnormalities.

Cognitive abnormalities, observed in five patients in isolation or together with muscle weakness, ranged from behavioural issues to severe global developmental delay. Risk of cognitive deficits among DMD patients is thought to be the result of the cumulative loss of dystrophin isoforms in the central nervous system during development [36]. According to this hypothesis, mutations in three subjects (#5, #19 and #20) were predicted to destroy most dystrophin isoforms. Nevertheless, in the other two subjects (subjects #1 and #18) only long isoforms were predicted to be affected. It is of particular note that four of the five cases presenting cognitive deficits showed highly skewed XCI patterns in blood independently of muscle symptoms. This could indicate that the central nervous system is more affected than muscle by reduced dystrophin expression due to biased XCI.

Skewed XCI favouring expression of the *DMD*-mutated X chromosome has been proposed as the main mechanism accounting for symptoms in symptomatic carriers without chromosomal rearrangements. However, results concerning the use of XCI as a prognostic marker are controversial. While some studies found that the great majority of symptomatic cases exhibited skewed profiles [20-22] others found that XCI is not a reliable measure to predict whether carrier female will develop symptoms [6,10,24]. Most women in the normal population present a random XCI pattern in peripheral blood. However, a skewed pattern is present in 8.8% of females, increasing up to 14.2% in adult women [29]. We found a distribution of XCI ratios in blood among control groups (asymptomatic carriers and non-carrier females) similar to those described in the normal adult population [29]. Regarding our group of symptomatic carriers, excluding those manifesting only myalgia, the number of skewed cases was 49.2% higher (mean XCI ratio 18% higher) than in asymptomatic carriers. In cases presenting skeletal muscle involvement, the extent of XCI skewing in blood tended to increase in line with the severity of symptoms (Figure 3B). However, assessing XCI through the *AR* methylation assay seems to have a poor prognostic value, since some subjects showing similar XCI patterns exhibited different phenotype severity. Furthermore, four subjects (#16, #18, #19 and #20) with highly skewed patterns presented no skeletal muscle involvement. When XCI analysis was performed on muscle DNA we observed no correlation between the extent of XCI skewing and clinical severity or proportion of dystrophin-negative fibres. As previously described [6,23], in most cases XCI profile in muscle differed significantly from that found in blood. This could reflect a biochemical and genetic normalization process in skeletal muscle [25,37,38], or tissue-specific differences in XCI or in the methylation status of the *AR* gene. In keeping with the latter hypothesis, in subject #3 the preferential expression of the *DMD* mutated allele correlated with the XCI ratio in blood but not with that in muscle (Figure 2). This case suggests that the methylation status of the *AR* gene in muscle may not reflect in all cases that of the *DMD* gene.

Soltanzadeh et al. [10] found that *DMD* deletions or duplications were significantly more associated with skewed XCI compared with point mutations suggesting a correlation between XCI and mutation class. Analysing our data, we found that skewed XCI was more frequent in patients with point mutations (5/7, 71.4%) compared with those carrying deletions or duplications (7/15, 46.7%). However, these differences do not reach statistical significance in a Fisher’s Exact Test suggesting that the *DMD* mutation class is not involved in the development of X inactivation skewness.

We found evidence of familial XCI skewing in 78.6% of analyzed families suggesting that in many cases this may be under genetic control. X inactivation is a complex process restricted to early embryogenesis in which an X chromosome is silenced at random depending on the stable expression of the cis-acting *XIST* gene [30]. Skewed X inactivation may be caused primarily by preferential silencing of one specific X chromosome due to genetic factors, or by chance due to the very limited number of precursor cells present at the moment of inactivation [39]. In X chromosome defects, skewing can also be secondary during development due to post-inactivation selection. Selection tends to preserve gene dosage: in balanced X:autosome translocations the normal X is generally inactivated while in X deletions the inactivated chromosome is the deleted X [39]. Mutations in the *XIST* gene promoter have been reported to cause primary non-random X-chromosome inactivation [31,32]. We analyzed the *XIST* promoter in all patients but no changes were detected. Other loci on the X chromosome have been associated with familial skewing of X inactivation, suggesting that factors other than *XIST* may control the primary choice of X chromosome or be secondarily involved in cell survival [40,41].

We consider that it could be clinically important to identify the genetic factors that alter random X inactivation in heterozygous females expressing X-linked recessive traits such as dystrophinopathy.

The reduced sample size and lack of systematic case collection limit the generalizability of the results.

## Conclusion

Our results demonstrate that the extent of XCI skewing is related to phenotype severity in symptomatic female carriers of dystrophinopathy. The methylation status of the *AR* gene in muscle may not reflect in all cases the methylation status of the *DMD* gene, conferring a poor prognostic value to the *AR* methylation assay for the assessment of XCI. Furthermore, skewed XCI is in many cases familial inherited.

## Abbreviations

DMD: Duchenne muscular dystrophy; BMD: Becker muscular dystrophy; XLCM: X-linked cardiomyopathy; XCI: X-chromosome inactivation; AR: Androgen receptor; MLPA: Multiple ligation-dependent probe amplification; DCM: Dilated cardiomyopathy; MR: Mental retardation; CK: Creatine kinase.

## Competing interests

All the authors stated that they have no interests which might be perceived as posing a conflict or bias.

## Authors’ contribution

JJM and PG designed the research, analyzed data and wrote the paper. JJM, MJR, EV and LGQ performed *DMD* molecular analysis in all the samples. AN, CP, MM, PSA, LGM, FM, MRQ, MR, JDM, JP, JC and MO performed the clinical characterization of the patients. CJM, ER, CJ, FM, AHL, EG and MO performed the pathological and immunohistochemical analyses of muscle biopsies. MB gave intellectual support. MO collaborated in the data analysis and in the writing of the paper. JJM and PG were primarily responsible for this work. All the authors read and approved the final manuscript.
